# Emerging Applications of Optical Coherence Tomography Angiography (OCTA) in neurological research

**DOI:** 10.1186/s40662-018-0104-3

**Published:** 2018-05-12

**Authors:** Liang Wang, Olwen Murphy, Natalia Gonzalez Caldito, Peter A. Calabresi, Shiv Saidha

**Affiliations:** 1 Department of Biology, Johns Hopkins University, Krieger School of Arts and Sciences, Baltimore, MD USA; 2 Division of Neuroimmunology and Neurological Infections, Johns Hopkins Hospital, 600 N. Wolfe St., Baltimore, MD 21287 USA

**Keywords:** Optical coherence tomography angiography, Neurology, Multiple sclerosis, Alzheimer’s disease, Optic neuropathy

## Abstract

**Purpose:**

To review the clinical and research value of optical coherence tomography angiography (OCTA) in the field of neurology.

**Methods:**

Current literature involving OCTA were reviewed through PubMed using the search terms “optical coherence tomography angiography”, with “multiple sclerosis”, “Alzheimer’s disease”, “optic neuropathy”, or other closely-related terms.

**Results:**

OCTA has been applied in research to advance our understanding of the pathobiology of neurological disorders. OCTA-derived blood flow and vessel density measures are altered in multiple sclerosis (MS), Alzheimer’s disease (AD), and various optic neuropathies (ON) in varying regions of the posterior segment vasculature of the eye. These emerging research findings support the occurrence of retinal vascular alterations across a host of neurological disorders and raise the possibility that vasculopathy can be clinically relevant since it contributes to the pathobiology of several neurological disorders.

**Conclusion:**

OCTA may be beneficial for neurological research. Additional investigations using OCTA in neurological disorders will help to further validate its clinical and research utilities in terms of characterizing the role of vasculopathy in neurological disorders.

## Background

Optical coherence tomography angiography (OCTA) enables visualization of the ocular microvasculature through non-invasive, high-resolution enface and depth resolved imaging [1]. First adapted from OCT and approved by the Food and Drug Administration (FDA) in 2015, OCTA provides structural and potentially functional information regarding the superficial and deep retina, as well as choroidal vasculature [2]. Vessel alterations including but not limited to neovascularization [3], capillary loss [4], non-perfusion [5], increase in vessel tortuosity [6], and decrease in relative flow rates [7] have been reported with OCTA in various ocular pathological conditions such as age related macular degeneration [8], macular telangiectasia [9], artery and vein occlusions [10, 11], diabetic retinopathy [12], and glaucoma [7]. Undetectable with commonly utilized ophthalmic tools such as traditional OCT and fundus photography, deeper, finer and more subtle vascular alterations are traditionally visualized through intravenous fluorescein angiography (IVFA), which requires the injection of a fluorescence dye [11]. However, leakage of the dye related to the breakdown in the blood-retinal-barrier has been reported to potentially obscure capillary loss, vessel distortion, and early neovascularization [13, 14]. While indocyanine green angiography (ICGA) can potentially better visualize deeper regions of the chorioretinal vasculature, this technique still employs contrast-based injections [15–17]. Since OCTA may allow these obscured structures to be more clearly distinguished, and non-invasively without the use of contrast agents, the development of OCTA represents a major contribution towards advancing ophthalmic imaging in ocular disease diagnosis, monitoring and treatment [17, 18]. While OCTA utilization has been growing in ophthalmic clinical practices, a potential application of OCTA in the field of neurology has also been under active investigation. Benefiting from OCTA’s depth imaging capabilities, pial vessels in the cerebral microvasculature of mouse models can still be visualized in the setting of intact scalps [19]. OCTA has also enabled rodent models of stroke and traumatic brain injury (TBI) to be studied in vivo. The real time analysis OCTA allows of the structural and potential functional alterations within the microvasculature have helped to further characterize disease complexes and more specifically, vascular components underlying the pathobiology of diseases, which could open up new therapeutic targets and avenues of clinical monitoring [20, 21]. Due to the lack of direct access, human cerebral microvascular alterations are difficult to non-invasively monitor in vivo. However, the brain can possibly be interrogated through the eyes since the vasculature of the retina and brain is similar in its anatomy and physiology [22, 23]. Furthermore, through the process of trans-synaptic degeneration, cerebral tissue injury may result in tissue degeneration of the optic nerves and retinal ganglion cells, which are supplied by the central retinal artery [24]. Thus, structural and functional changes in the optic nerve and retina may hypothetically result in ocular vascular alterations. Using OCTA, non-invasive in vivo studies of neurological disorders through the human eye have emerged for multiple sclerosis (MS) [25–28], Alzheimer’s disease (AD) [29–31], and various optic neuropathies (ON) [32–36].

### Algorithms and analysis

**Fig. 1:**
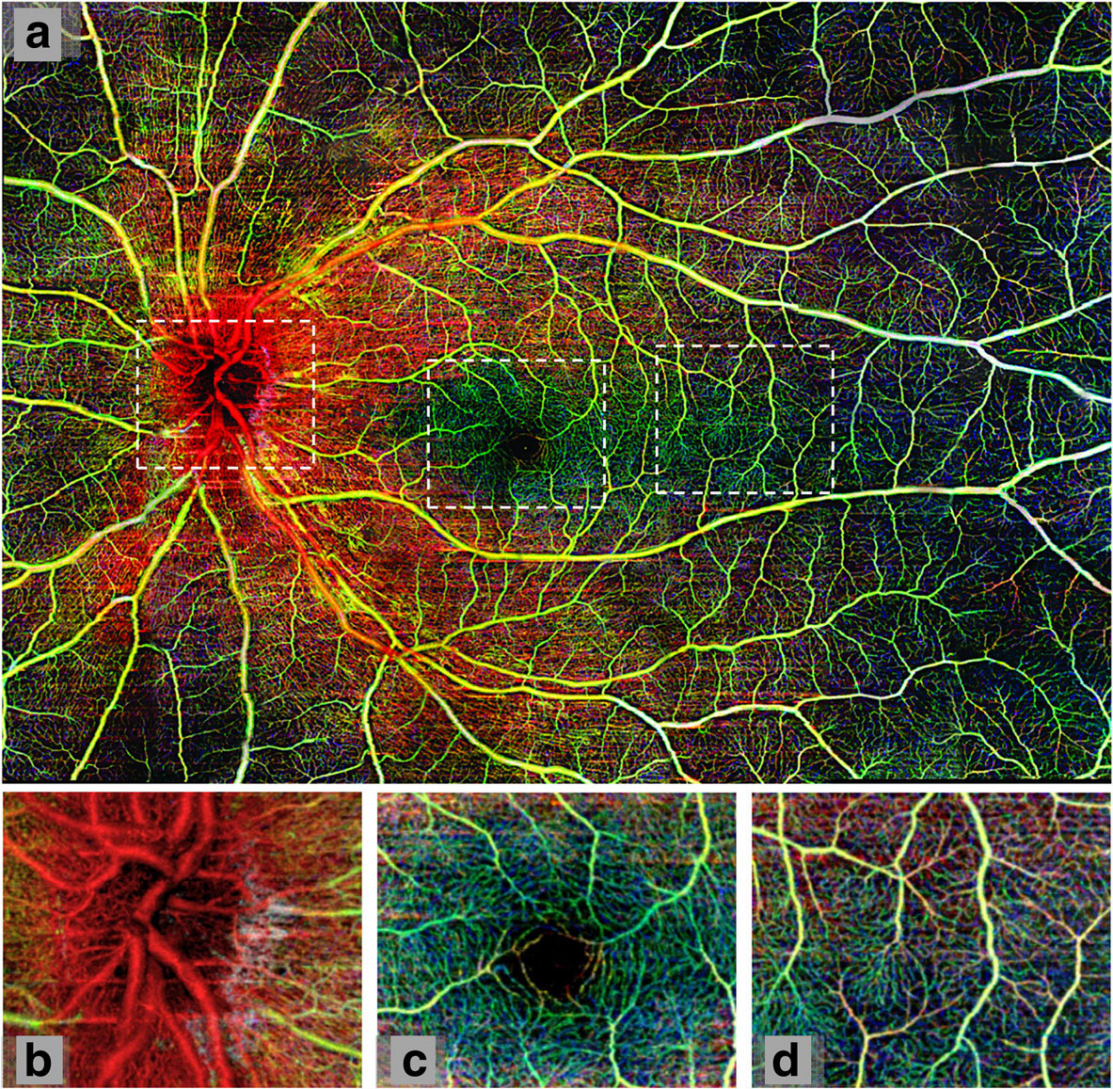
Wide field montage of healthy retinal microvasculature. Using Cirrus™ optical coherence tomography angiograph with optical microangiography, 42 scans were taken and combined to create a 12 × 16 mm^2^ image (panel **a**). The images are portrayed in different colors representing varying retinal depth. Red depicts the retinal nerve fiber layer, green the ganglion cell and inner plexiform layers, and blue the inner nuclear and outer plexiform layers. White boxes outline (**b**) optic nerve head, (**c**) fovea, and (**d**) temporal region. (Reprinted from Zhang et al. Wide-field imaging of retinal vasculature using optical coherence tomography-based microangiography provided by motion tracking. JBO, 20, 066009 (2015), DOI: 10.1117/1.JBO.20.6.066008) [43]

Adapted from traditional OCT, a powerful non-invasive imaging tool for visualizing and segmenting the discrete layers of the retina, OCTA enables high-speed imaging acquisition of the retinal vasculature for angiography. Analysis of the retinal vasculature can be completed through a number of algorithms. Ultrahigh-sensitive optical microangiography (OMAG, Cirrus HD-OCT 5000™, Carl Zeiss Meditec. Inc) [37], split-spectrum amplitude decorrelation angiography (SSADA, Optovue RTVue XR Avanti™, Optovue Inc., Fremont, CA) [38], OCT Angiography Ratio Analysis (OCTARA, Topcon DRI OCT Triton Swept source OCT™, Topcon, Japan), and full spectrum amplitude decorrelation algorithm (FS-ADA, Spectralis OCT2 module prototype™, Heidelberg Engineering, Germany) [39] have been commonly used in OCTA studies. These algorithms produce three dimensional, 3 × 3 mm^2^ A lines in cross-sectional images or 1 pixel in the enface image in less than 3 s. The A scan (line in the cross-sectional image, 1 pixel in the enface image) has a scan rate of 68 kHz with OMAG, 70 kHz with SSADA, 100 kHz with OCTARA, and 85 kHz with FS-ADA. The A scans make up B scans. The B scan is taken four times in the same region with OMAG and OCTARA. Two consecutive B scans are taken with SSADA and only one B scan is required with FS-ADA [40]. Images with a field of view larger than 6 × 6 mm^2^ can also be acquired using commercially available systems, but they often have a lower lateral resolution with individual A-scans covering the scan areas. Images with larger fields of view and relatively higher lateral resolution can be acquired using the high speed Swept Source (SS) OCTA system [41, 42] or through wide-field montages (Fig. 1) [37].

**Fig. 2:**
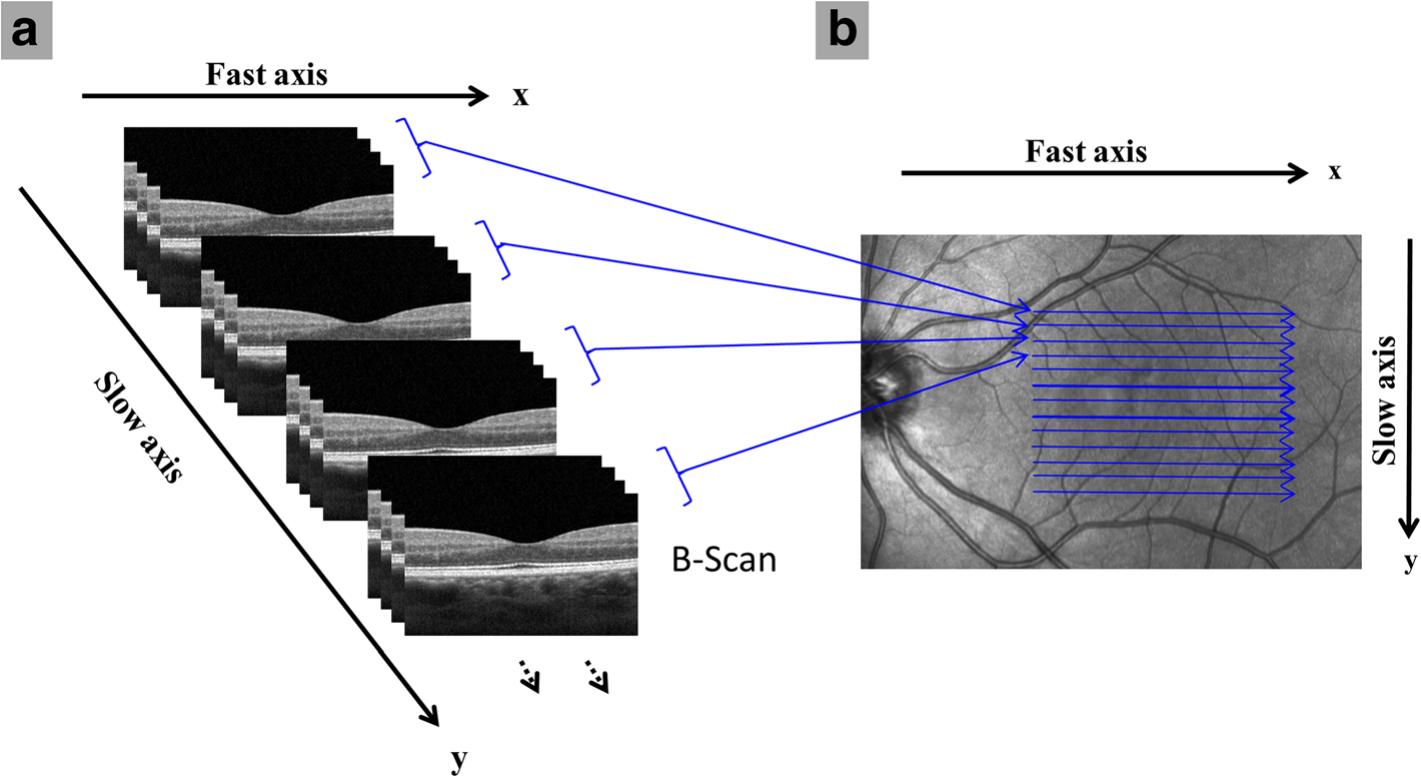
General scanning protocol for optical coherence tomography angiography (OCTA). (**a**) Repeated B-scans are taken on the “x” fast axis at each of the “y” slow scan axis points to detect relative flow signal. (**b**) Top view of the same general scan pattern as (**a**) with repeated B-scans taken on the fast axis “x” along each “y” location of the slow axis. Sample scans were obtained with Angiovue™ OCTA (**a**, **b**)

To detect red blood cell movement in OCT images, repeated B-scans are taken at the same cross-section of the same location with similar scanning methods used in other OCTA systems (Fig. 2) [2, 42, 43]. Changes in the phase and intensity of the OCT signal can then be detected through the motion of red blood cells. OMAG utilizes both of the potential changes to generate angiograms with comparably high image contrast and vessel connectivity. Alternatively, SSADA utilizes the signal intensity through decorrelation values between consecutive B-scans where increased red blood cell movement resulted in increased decorrelation values [2]. Thus, areas of low decorrelation may indicate regions of non-perfusion and/or vessel loss. This algorithm decreases the effects of phase noise that may occur especially when the light source is unstable. The signal is also further processed to improve vessel detection and remove noise introduced by motion [38]. Instead of decorrelation values, OCTARA uses intensity ratio analyses, which helps increase the axial resolution by not splitting the spectrum. On the other hand, FS-ADA can be used to analyze blood flow through motion contrast [40].

Owing to the fact that OCTA has traditional OCT function that can be used to acquire information on retinal structure, multiple layers can be identified so that the vasculature in the corresponding layers can be segmented [44]. For example, in Optovue™, the OCT scan can be segmented into a number of composite layer regions, including the inner retina (from ganglion cell layer to inner plexiform layer), middle retina (from inner nuclear layer to outer plexiform layer), outer retina (from outer nuclear layer to external limiting membrane), choriocapillaris, and choroid [8]. These regions on the OCTA can be analyzed for a number of vascular features. For instance, the presence of neovascularization [45], an increase in tortuosity [6], and areas of capillary loss [46] can be qualitatively analyzed. Additional features such as the area of the foveal avascular zone (FAZ), parafoveal region, regions of non-perfusion and relative density can be quantitatively determined either through the fractal analysis or pixel counting methods. The flow rate is the average decorrelation value of sequential B-scans on OCTA, which can be used as a surrogate for blood flow rates [47]. Furthermore, Optovue™ includes an analysis package in its system that automatically analyzes the FAZ and vessel density in different retinal sub-regions.

### Healthy eyes

**Fig. 3:**
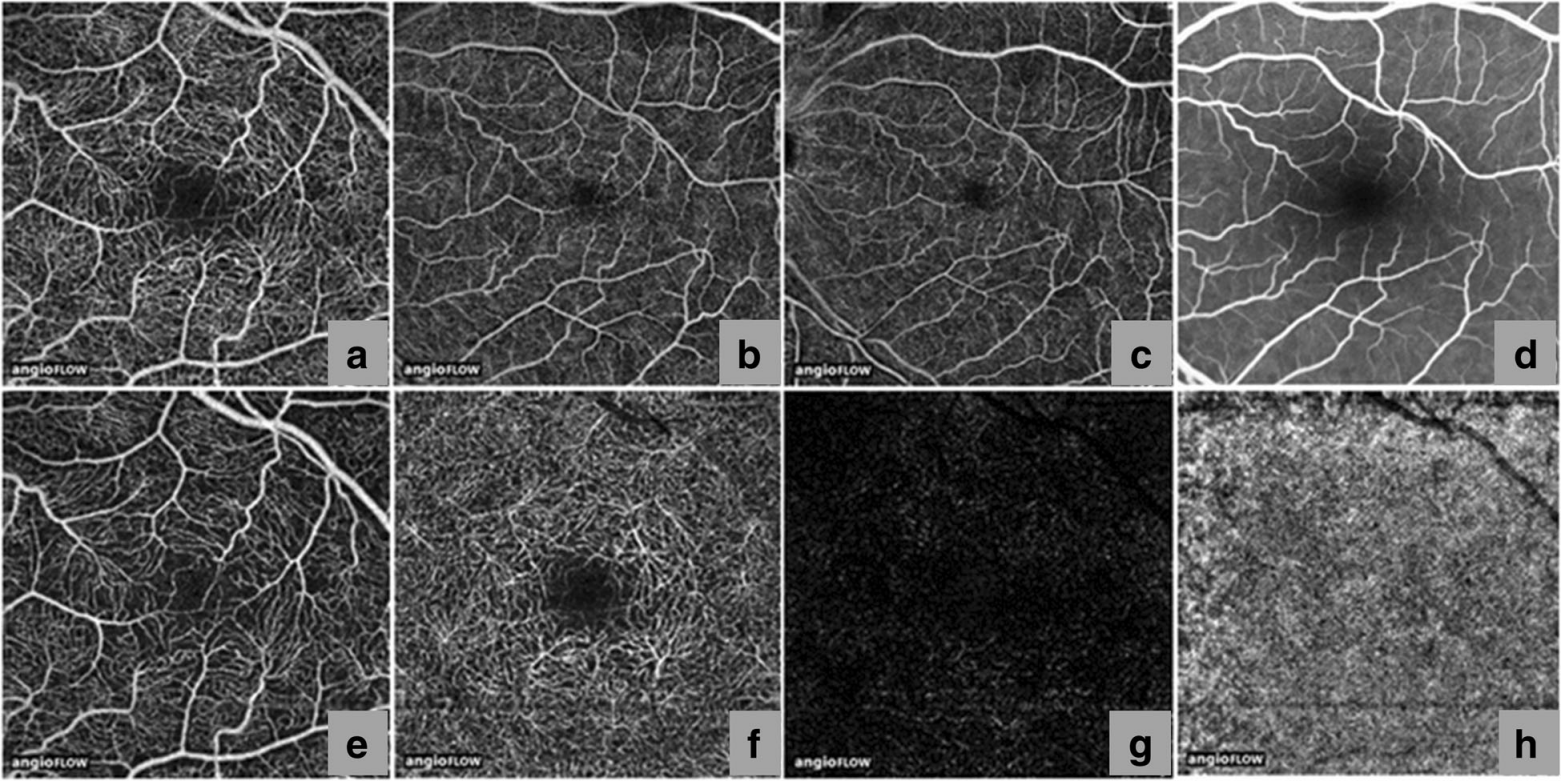
Anatomy of the posterior segment vasculature. Using Angiovue™ optical coherence tomography angiography (OCTA), the vascular plexus from the internal limiting membrane to the Bruch’s membrane of a healthy subject was non-invasively visualized in 3 × 3 mm (**a**), 6 × 6 mm (**b**), and 8 × 8 mm (**c**) angiograms. Intravenous Fluorescein Angiography (IVFA) cropped to 8 × 8 mm (**d**) shows less microvasculature detail than that of OCTA angiogram (**a**-**c**). The inner retinal vascular plexus is further separated into the superficial inner vascular plexus (**e**), which supplies the retinal nerve fiber layer and ganglion cell layer and the deep inner vascular plexus (**f**), which supplies the inner plexiform layer, inner nuclear layer, and outer plexiform layer [76]. No distinct vasculature can be detected in the outer retina (**g**) and choriocapillaris (**h**) using 3 × 3 mm OCTA. (Reprinted and modified from de Carlo et al. A review of optical coherence tomography angiography (OCTA), Int J Retina Vitreous, 1,5 (2015), DOI: 10.1186/s40942-015-0005-8) [76]

The eye enables one of the dominant methods of perception by focusing light stimuli through the anterior segment onto the posterior segment, which includes the photosensitive cells in the retina that are responsible for converting light stimuli into electrochemical impulses, which are subsequently sent to the brain through the optic nerve [24]. Using OCTA, the vascular plexuses of the inner retina, outer retina, and choriocapillaris can be non-invasively visualized. Supplying the retina, one of the regions of the body with the highest metabolic demand, the inner retinal vascular plexus is further separated into the superficial vascular plexus (SVP), which supplies the retinal nerve fiber layer, andganglion cell layer, and the deep vascular plexus (DVP), which supplies the inner plexiform layer, inner nuclear layer, and outer plexiform layer (Fig. 3) [48]. In healthy eyes, OCTA cross sectional images show clear retinal and choroidal layers with relatively even blood flow, corresponding with en face angiograms that show healthy retinal and choroidal layers with dense and well connected vasculatures [38]. The en face angiograms also show normal avascular regions in the vitreous above the inner limiting membrane and in the outer retina between the outer plexiform layer and Bruch’s membrane. OCTA findings of microvascular structure, including capillary patterns of the perifoveal and peripapillary regions have been verified through comparison to examined *post-mortem* tissue in histology studies [14].

OCTA findings in healthy volunteers have been critical for forming a solid foundation for the normative retinal and choroidal microvasculature, determining when abnormalities are present, and will ultimately play a central role in helping to guide future disease characterization and diagnosis. The mean relative parafoveal flow (flow index) and relative vessel coverage (decorrelation values) have been reported to significantly decrease with normal aging, while the FAZ area (determined by pixel counting) significantly increases in size with normal aging [49]. The correlation between vessel and perfusion loss with increasing age in healthy vasculatures have been reported in prior studies using blue field stimulation [50], magnetic resonance imaging [51], and IVFA [52]. However, analyses of the FAZ determined by fractal analysis, as opposed to pixel counting, did not find significant changes in the FAZ with age, which might be partially due to unadjusted magnification error [53]. However, fractal dimension showed a relatively denser DVP in comparison to the SVP in relation to age-related changes [18, 54]. These findings are supported by previous research that analyzed human donor eyes through confocal microscopy in the perifoveal region [55]. The FAZ area, which varies in size from person to person has been reported to be on average 0.32 ± 0.11 mm^2^ in size through pixel counting on angiograms generated with SSADA in a study of healthy volunteers (*n* = 144, age range 10–79 years) [56].

### Multiple sclerosis

Multiple Sclerosis (MS) is a chronic inflammatory demyelinating disorder of the central nervous system (CNS) where neurodegeneration primarily occurring because of inflammation is thought to be the principal substrate underlying disability. *Post-mortem* studies demonstrate that up to 99% of MS patients exhibit demyelinating plaques within their optic nerves, making optic nerve involvement a ubiquitous part of the MS disease process [57, 58]. Optic neuritis (ON) is the initial manifestation of MS in approximately 25% of patients and occurs in approximately 50% of patients at some point in the disease course [28]. Since available treatments generally target the autoimmune and inflammatory aspects of MS and vary in effectiveness across patients [27], sensitive biomarkers for monitoring disease progression and therapeutic efficacy are desperately needed. OCT-derived retinal measures have emerged for this purpose, with the retina representing the most accessible component of the CNS. OCT identified thinning of the retinal nerve fiber layer (RNFL) and composite of the ganglion cell and inner plexiform layers (GCIP) in particular have become biomarkers of global CNS neurodegeneration in MS [59].

**Fig. 4:**
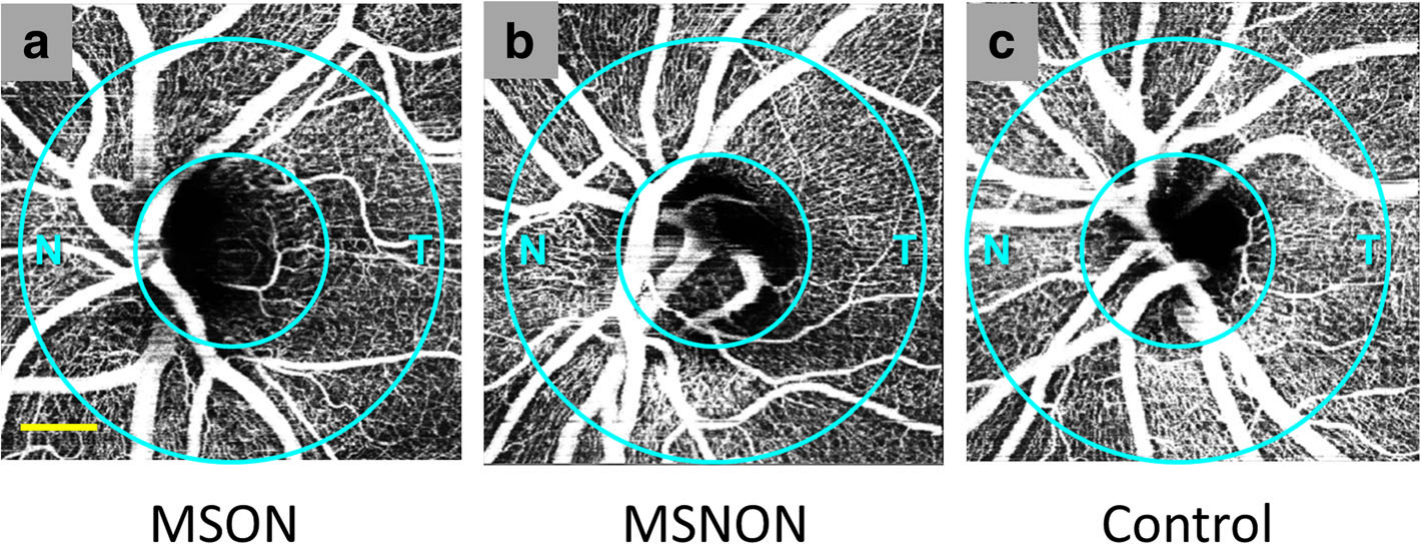
Optical coherence tomography angiography (OCTA) of the optic nerve head (ONH) in a representative Multiple Sclerosis (MS) patient. As determined with split-spectrum amplitude decorrelation angiography (SSADA), images (*N* = nasal, T = temporal) show apparent qualitative reduction of the ONH microvascular density in the peripapillary area (between circles) predominantly in the temporal region in both MS eyes with a history of ON (MSON) (**a**) and MS eyes without a history of ON (MSNON) (**b**) eyes in comparison to a healthy control example (**c**). Bar = 0.5 mm

OCTA now shows utility in detecting and studying damage to the retinal vasculature in MS. The parafoveal and optic nerve head (illustrated in Fig. 4) flow index, a representation of relative blood flow velocity in the vasculature, was determined to be significantly lower in MS eyes with a history of ON (MSON) in comparison to eyes without a history of ON (MSNON), as well as relative to eyes in healthy controls [25, 28] (Table 1). Furthermore, significant vessel reduction in the superficial and deep retinal vascular plexuses in the parafoveal area was found in both MSON and MSNON eyes as compared to healthy control eyes [26, 27]. Retinal vascular density was correlated with retinal layer thicknesses (RNFL, GCIP, total macular volume and inner nuclear layer) and increased disability in MS patients as determined through Expanded Disability Status Scale scores [27].

The etiology underlying the decrease in blood flow and density of the retinal vasculature in MS patients remain unclear. Accumulating evidence has determined that inflammation leads to loss of neurons in the inner retina. The reduced number of neurons, resulting in reduced metabolic demand, may result in reduced blood supply provided by the vessels of the SVP, which in turn supplies the DVP through anastomoses [25, 26]. Inflammation is thought to lead to mild alterations in vascular function, including endotheliopathy, and postulated to result in decreased perfusion and neuronal damage [60], although this pathologic disease mechanism remains to be definitively demonstrated. Interestingly, studies of the cerebral microvasculature in MS have revealed possible hypoperfusion in the normal appearing gray matter and white matter [61, 62]. A recent cross-sectional study of the Retinal Function Imager, an ophthalmic multimodal imaging modality, in relapsing remitting MS (RRMS) (*n* = 17) revealed reductions in retinal arteriolar and venular blood flow velocities as compared to healthy controls, although determinations with respect to underlying mechanisms of the observed findings or their relationships to ON history were not possible [63]. In MS, it is also recognized that elevations in hypoxia inducible factors (indicating vascular compromise) occur in regions of neurodegeneration [64]. Future studies employing easily performed OCTA with larger sample sizes, longitudinal follow up, and more focus on potential correlations to other MS biomarkers in the CNS will help further characterize aberrations in the retinal vasculature in MS and provide insights into MS disease mechanisms and even novel therapeutic strategies.

### Alzheimer’s disease

Alzheimer’s disease (AD), the most common cause of dementia [65], is a progressive neurodegenerative disorder that is more common with aging and characterized by an accumulation of misfolded protein, in particular amyloid-beta (AB) and neurofibrillary tangles (NFTs) [29]. Prior to AD onset, patients often have mild cognitive impairment (MCI), during which cognitive function declines, but patients have sufficient cognitive function to perform their activities of daily living [66]. AD has been associated with a number of vascular risk factors including stroke, diabetes mellitus, atherosclerosis, and hypertension [67]. Moreover, cerebral hypoperfusion, increased cerebral vascular tortuosity, and decreased vascular density have been reported in AD patients [29, 68], which may indicate the presence of a vasculopathy in the pathogenesis of AD. This probable disease mechanism, while difficult to analyze directly due to the location of the brain within the skull, may be investigated through the retinal microvasculature. The retinal and cerebral vasculatures share similar embryonic, physiologic, and anatomic features [30, 63]. Furthermore, similar to the brain, AB accumulation was found in the retina in AD [69]. Using OCT, thinning of the GCIP in the retina was suggested to be a potential biomarker of neurodegeneration and disease severity in AD [70].

**Fig. 5:**
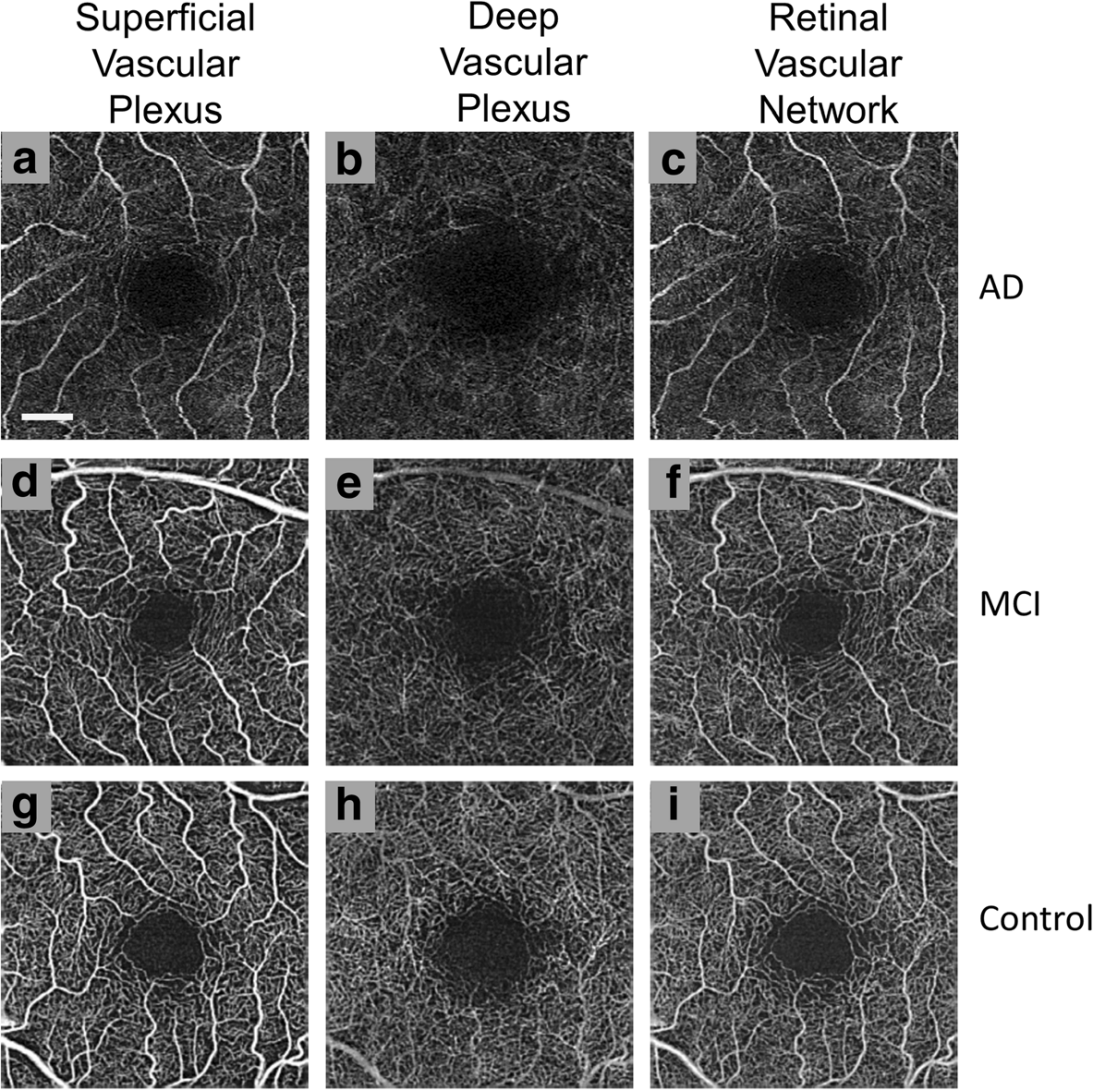
Optical coherence tomography angiography (OCTA) of the parafoveal region in Mild Cognitive Impairment (MCI) and Alzheimer’s Disease (AD). As determined using Cirrus ™, retinal microvasculature for patients with AD (**a**, **b**, and **c**), MCI (**d**, **e**, and **f**), and healthy controls (**g**, **h**, and **i**) are shown. For the large vessels, no significant differences in density were observed, but some degree of increased tortuosity was seen in the superficial vascular plexus (SVP) (**a**) in comparison to normal controls (**g**). The microvasculature of the deep vascular plexus (DVP) (**b**) had a significant decrease in density with a visually larger foveal avascular zone when compared with normal controls (**h**) and MCI patients (**e**). The overall retinal vascular network contains both the SVP and DVP (**c**, **f**, and **i**). Bar = 0.5 mm [31]. (Images provided courtesy of Dr. Hong Jiang, MD, PhD of Bascom Palmer Eye Institute at the University of Miami)

Further assessments of the individual vessel plexuses of the retina are possible through OCTA. All regions of the SVP and DVP in AD patients were found to exhibit significantly lower retinal vascular density when compared to healthy controls (Fig. 5) [29, 31]. The loss of retinal vessel density could be an indication of the increased accumulation of AB in the retina [69], which can theoretically confine vascular endothelial growth factor (VEGF) within amyloid plaques and potentially reduce angiogenesis [29]. Furthermore, significant enlargement of the foveal avascular zone (FAZ) formed by the retinal plexuses was observed using OCTA in AD, which is a potential sign of ischemia and in itself may contribute to reducing retinal vessel density [29]. However, only DVP loss has been found to be significantly correlated with GCIP thinning in AD. This may be related to the relatively larger vessels within the SVP that may be less sensitive to disease progression in comparison to those of the DVP [31]. The retinal microvasculature may be a sensitive marker for cerebral microvasculature alterations, which could be useful for monitoring the progression of AD. In summary, OCTA analysis of the retinal microvasculature revealed significant loss of vessel density in AD patient eyes in comparison to eyes from normal controls and patients with MCI [31]. Future studies with larger patient cohorts may help to establish the retinal vasculature, as imaged by OCTA, as a biomarker of AD, predictor of conversion from MCI to AD, and a tool for investigating the effectiveness of putative AD treatments.

### Optic neuropathy

#### Anterior ischemic optic neuropathy

Characterized by severe vision loss due to ischemia of the small vessels supplying the anterior portion of the optic nerve head, anterior ischemic optic neuropathy (AION) can be classified as arteritic (AAION) or non-arteritic (NAION) [35]. Accounting for up to 15% of AION cases, AAION is caused by inflammation within arteries primarily as a result of underlying giant cell arteritis (temporal arteritis) [32]. NAION is the more common form of AION and accounts for approximately 85% of AION cases. NAION is considered a form of small vessel disease, which usually occurs in patients with various risk factors such as hypertension, diabetes, or dyslipidemia. These factors are unrelated to inflammation [32]. Furthermore, there is no consistently effective treatment method to prevent irreversible vision loss in NAION. While OCTA cannot differentiate between AAION and NAION, the regions of non-perfusion with ischemic boundaries can be clearly identified on OCTA of the optic disk [32].

**Fig. 6:**
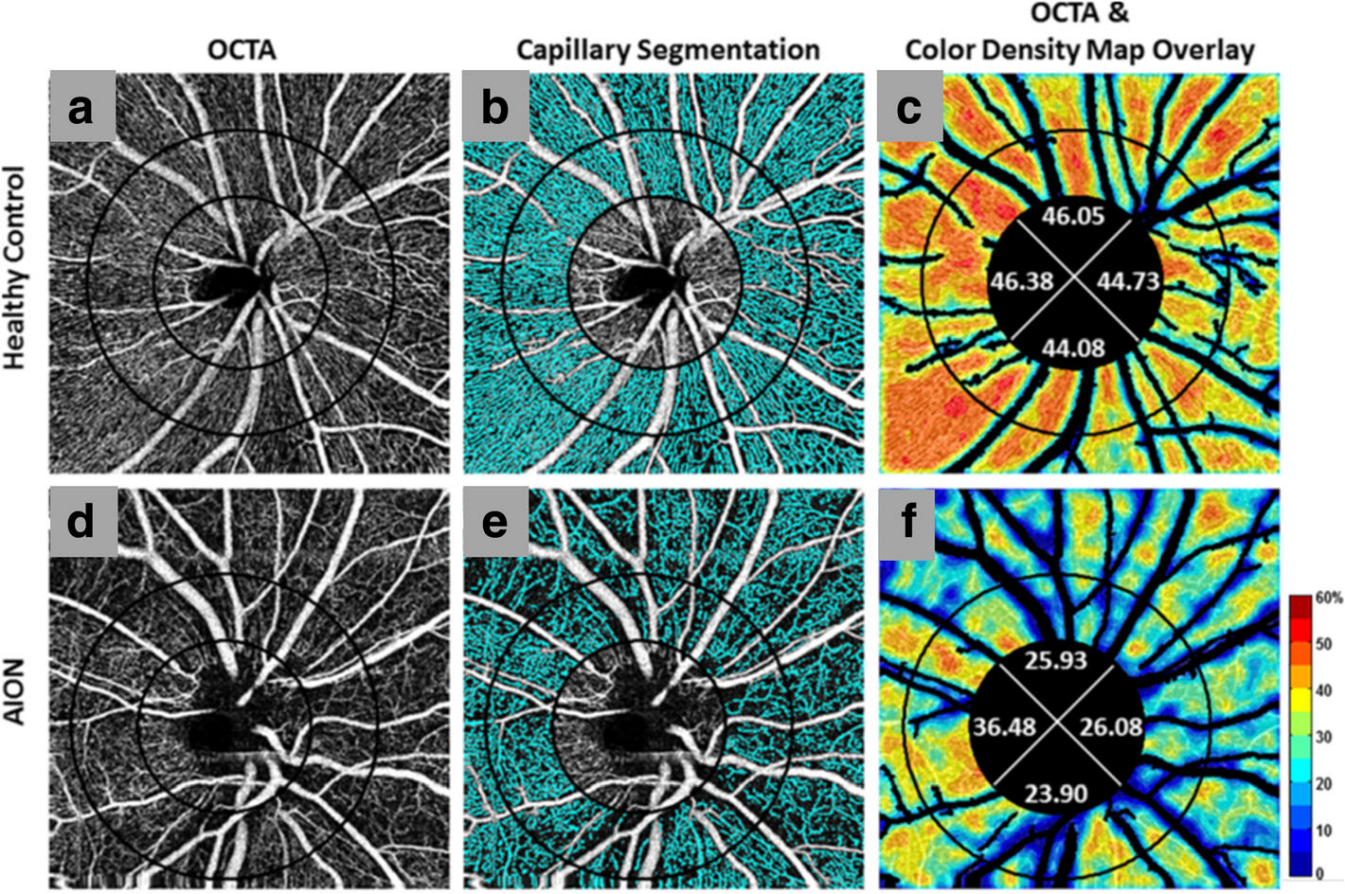
Optical coherence tomography angiography (OCTA) with peripapillary capillary density maps in Non-arteritic Anterior Ischemic Optic Neuropathy (NAION). Images were derived using Angiovue™ with SSADA and they depict optic disc-centered angiograms (first column) (**a**, **d**) with perfused microvessels marked in cyan (second column). The major vessels were not included in the capillary segmentation in panels **b** and **e**. Original OCTA overlaid with corresponding color-coded density map (legend: higher density % up to 60% = redder) and capillary density percentage for four quadrants (third column) (**c**, **f**). Patients with NAION had a smaller perfusion region when compared with normal controls as seen with the visible loss of microvessels (cyan) in NAION (**e**) in comparison to healthy controls (**b**) and the decreased capillary density percentages (less red overall) for all quadrants in NAION (**f**) in comparison to healthy controls (**c**). (Reprinted from Fard et al. Pattern of peripapillary capillary density loss in ischemic optic neuropathy compared to that in primary open-angle glaucoma. PLoS One, 13:e0189237 (2018), DOI: 10.1371/journal.pone.0189237) [33]

OCTA has shown promise in monitoring NAION progression through sectorial peripapillary capillary density reduction [32, 33] (Fig. 6) and increased non-perfusion area percentages. The non-perfusion area was shown to be significantly increased in NAION eyes in comparison to healthy control eyes, and correlates with the severity of peripheral visual field loss [35] and central vision impairment (as assessed by best corrected visual acuity) [36]. Furthermore, significant reduction of the RNFL and GCIP [71] in the macular region have been reported in NAION eyes assessed by OCT. Since decreased optic disc perfusion detected via OCTA shows correlation with GCIP and RNFL thicknesses in patients with glaucoma, OCTA might also be an ideal candidate for monitoring NAION progression and identify potential OCTA-based biomarkers for NAION [35, 36].

#### Other optic neuropathies

Primarily affecting males between the ages of 15 and 35, Leber’s hereditary optic neuropathy (LHON) is a rare genetic disorder inherited through mitochondrial DNA [72]. LHON typically presents as monocular optic atrophy [34] that develops into bilateral central visual field defects within weeks to months after initial symptom onset. The characteristic microangiography of LHON accompanied by RNFL thinning has been non-invasively demonstrated by OCTA as increased tortuosity and decrease in observable vessels in the peripapillary microvasculature. The reduced visibility of microvessels indicates thinning of the vasculature with decreased vessel density, which could theoretically relate to lowered metabolic demand in the setting of RNFL/axonal degeneration. Alternatively, it is possible that the pathobiology of LHON may directly include destruction of the microvessels, although this remains to be elucidated [34]. Further larger studies using OCTA may help to delineate the role of microvascular aberrations as part of the LHON disease process.

**Table 1:** Summary of significant optical coherence tomography angiography findings in neurological disorders *

Author	Parameter	Disorder	*N* (eyes)	Measurement	Compare	*N* (eyes)	Measurement	% Difference
Wang et al. (2014) [25]	*flow index - ONH*	MSON	14	0.140 ± 0.020	HC	21	0.160 ± 0.010	−14.3%
Spain et al. (2017) [28]	*flow index - ONH*	MSON	25	N/A	HC	55	N/A	−14.7%
Feucht et al. (2017) [26]	*vessel density % - SVP*	MSON	16	50	HC	100	54	−4.0%
	*vessel density % - SVP*	MSON	16	50	MSNON	56	53	−3.0%
	*vessel density % - DVP*	MSON	16	62	HC	100	64	−2.0%
	*vessel density % - DVP*	MSON	16	62	MSNON	56	64	−2.0%
Lanzillo et al. (2017) [27]	*vessel density % - parafovea IS*	MSON	23	50.96 ± 5.33	MSNON	77	51.71 ± 5.82	−1.5%
	*vessel density % - macula*	MS	100	48.71 ± 4.44	HC	92	53.08 ± 3.31	−9.0%
Bulut et al. (2017) [29]	*area (mm* ^*2*^ *) - FAZ*	AD	26	0.47 ± 0.18	HC	26	0.33 ± 0.08	29.8%
	*vessel density % - macula*	AD	26	45.50 ± 3.85	HC	26	48.67 ± 3.29	−7.0%
Jiang et al. (2017) [31]	*vessel density - SVP*	AD	12	1.68	HC	21	1.74	−3.6%
	*vessel density - DVP*	AD	12	1.65	HC	21	1.72	−4.2%
	*vessel density - DVP SN*	MCI	19	1.54	HC	21	1.62	−5.2%
Fard et al. (2018) [33]	*vessel density % - PC*	NAION	31	30.1 ± 6	HC	77	42.3 ± 2.3	−40.5%
Song et al. (2017) [36]	*vessel density % - PC*	NAION	41	52.07 ± 7.68	HC	30	58.68 ± 3.16	−12.7%
Ling et al. (2017) [35]	*nonperfusion area % - ONH*	NAION	21	17.84 ± 6.18	HC	19	8.61 ± 1.65	51.7%

*Abbreviations*: *ONH* = optic nerve head; *SVP* = superficial vascular plexus; *DVP* = deep vascular plexus; *IS* = inferior sector; *FAZ* = foveal avascular zone; *SN* = superior nasal quadrant; *PC* = peripapillary capillaries; *MSON* = *Disorder:* multiple sclerosis with a history of optic neuritis; *MS* = multiple sclerosis with and without history of optic neuritis; *AD* = Alzheimer’s disease; *MCI* = mild cognitive impairment; *NAION* = non-arteritic anterior ischemic optic neuropathy; *HC* = *Compare:* healthy controls; *MSNON* = multiple sclerosis without a history of optic neuritis

*Listed comparisons were significantly different (*P* < 0.05)

Resulting from elevated intra-cranial pressure that may be idiopathic in etiology or secondary to numerous causes including but not limited to intracranial masses for example, papilledema –is swelling of the optic discs – that usually occurs bilaterally. Vision loss is uncommon in early papilledema, but decreased visual acuity and visual field loss is often profound in advanced papilledema [73]. Using OCT, papilledema has also been shown to be associated with a significant increase in disc volume due to overall swelling, but also degeneration of the inner retinal layers in the setting of chronic papilledema [74]. OCTA demonstrated an increased visibility of the peripapillary vascular network in chronic papilledema, which reveal an increased diameter of vessels along with an increase in vessel density [34]. Additional studies with a quantitative measurement of the microvasculature for perfusion density will be necessary to further characterize the role of vascular alternations in the progression of papilledema.

## Conclusions

OCTA is an advanced ophthalmic imaging technique that can non-invasively generate angiograms with depth information for the posterior segment of the eye. The subsequent measures of retinal vascular structure and indirect blood flow enable the detection of alterations in the structure and function of the vasculature in the retina, including the microvasculature that can be qualitatively and quantitatively analyzed. It is worthwhile to note limitations of the OCTA, including the lack of an industry standard for image processing, segmentation of vessel layers, and quantitative analysis. Furthermore, anatomical variables such as individual differences in blood flow and position of vessels may cause variability between subjects, both especially for qualitative and quantitative studies. Nevertheless, while the field of view is relatively small in comparison to other ophthalmic techniques such as FA, ICGA, and RFI [11, 17, 75], significant findings have been identified in a number of potentially sight-threatening ophthalmic disorders [76]. Capable of non-invasively imaging microvascular features that may be obscured or undetectable in other ophthalmic imaging techniques, OCTA utilization is growing across a host of neurological disorders (including cerebrovascular diseases) and contributing towards advancing our understanding of operative disease mechanisms as well as the potential identification of novel biomarkers, which could collectively lead to novel therapeutic targets/strategies in the future. Blood flow or vessel density was reported to be decreased in MS, AD, and ON in various regions of the posterior segment vasculature. These emerging research findings suggest a role for vascular alterations in the pathobiology of these diseases, although it is unclear if and to what magnitude these changes may contribute to disease progression. Further investigations with larger sample sizes, assessment of greater regions of the vasculature, and additional OCTA parameters may help to characterize these neurological disorders and determine the true utility of OCTA in the field of neurology.
